# Altered resting-state functional activity in posttraumatic stress disorder: A quantitative meta-analysis

**DOI:** 10.1038/srep27131

**Published:** 2016-06-02

**Authors:** Ting Wang, Jia Liu, Junran Zhang, Wang Zhan, Lei Li, Min Wu, Hua Huang, Hongyan Zhu, Graham J. Kemp, Qiyong Gong

**Affiliations:** 1 Department of Medical Information Engineering, School of Electrical Engineering and Information, Sichuan University, Chengdu, P. R. China; 2 Huaxi MR Research Center (HMRRC), Department of Radiology, West China Hospital of Sichuan University, Chengdu, P. R. China; 3 Neuroimaging Center, University of Maryland, College Park, Maryland, USA; 4 Laboratory of Stem Cell Biology, State Key Laboratory of Biotherapy, West China Hospital of Sichuan University, Chengdu, P. R. China; 5 Magnetic Resonance and Image Analysis Research Centre (MARIARC) and Institute of Ageing and Chronic Disease, University of Liverpool, United Kingdom; 6 Department of Psychology, School of Public Administration, Sichuan University, Chengdu, P. R. China

## Abstract

Many functional neuroimaging studies have reported differential patterns of spontaneous brain activity in posttraumatic stress disorder (PTSD), but the findings are inconsistent and have not so far been quantitatively reviewed. The present study set out to determine consistent, specific regional brain activity alterations in PTSD, using the Effect Size Signed Differential Mapping technique to conduct a quantitative meta-analysis of resting-state functional neuroimaging studies of PTSD that used either a non-trauma (NTC) or a trauma-exposed (TEC) comparison control group. Fifteen functional neuroimaging studies were included, comparing 286 PTSDs, 203 TECs and 155 NTCs. Compared with NTC, PTSD patients showed hyperactivity in the right anterior insula and bilateral cerebellum, and hypoactivity in the dorsal medial prefrontal cortex (mPFC); compared with TEC, PTSD showed hyperactivity in the ventral mPFC. The pooled meta-analysis showed hypoactivity in the posterior insula, superior temporal, and Heschl’s gyrus in PTSD. Additionally, subgroup meta-analysis (non-medicated subjects vs. NTC) identified abnormal activation in the prefrontal-limbic system. In meta-regression analyses, mean illness duration was positively associated with activity in the right cerebellum (PTSD vs. NTC), and illness severity was negatively associated with activity in the right lingual gyrus (PTSD vs. TEC).

Posttraumatic stress disorder (PTSD) is a psychiatric illness caused by traumatic events, characterized by traumatic event re-experiencing (e.g. flashbacks), avoidance of trauma-related events, hyperarousal (e.g. hypervigilance), and negative cognitions and mood^1^. Present understanding emphasizes the contribution of deficient cognitive and emotional processes to the symptoms of PTSD^2^,^3^. This implicates a variety of brain regions including the amygdala, prefrontal cortex, temporal cortex, insula, thalamus, anterior cingulate cortex (ACC) and hippocampus^4^,^5^,^6^. Neurocircuitry linking the increased activity of limbic regions such as amygdala and insula and the decreased medial prefrontal activation may also contribute to the anxiety and emotional dysregulation in PTSD^4^,^7^.

Multiple neuroimaging modalities such as functional magnetic resonance imaging (fMRI), single-photon emission computed tomography (SPECT), and positron emission tomography (PET) have been employed to investigate the aforementioned altered brain activities in PTSD. In general terms, fMRI makes use of the blood oxygen level-dependent (BOLD) signal to show patterns of activity in the brain^4^, either in response to specific tasks or in the so-called resting state; two analysis methods, amplitude of low-frequency (0.01–0.08 Hz) fluctuation (ALFF)^8^ and regional homogeneity (ReHo)^9^, have been used to quantify patterns of fMRI resting-state activity. In addition, both regional cerebral blood flow (rCBF)/PET/SPECT and glucose metabolism (rCMglu)/PET can be used to visualize the activity of specific brain regions, and these have proved useful in studying PTSD^4^,^10^. The four different techniques of ALFF^11^, ReHo^9^, rCBF^12^, and rCMglu^13^ have generally been considered to reflect regional spontaneous neuronal activity in a similar manner, lending themselves to similar quantitative interpretation in terms of brain physiology; it therefore makes sense to combine them to explore the neural activity patterns of PTSD^14^,^15^,^16^,^17^.

Resting-state neuroimaging, which evaluates regional interactions that occur when a subject is not performing an explicit task, has proved an informative and reliable research tool^18^ which can provide insights into the pathophysiology of PTSD. Several studies have examined resting brain activity in PTSD^12^,^19^,^20^,^21^,^22^,^23^,^24^,^25^,^26^,^27^,^28^, revealing significantly different spontaneous activity in the cerebral cortex (e.g. superior temporal gyrus, medial prefrontal cortex (mPFC), inferior parietal lobule and middle occipital gyrus), the limbic regions (e.g. the amygdala, hippocampus, insula, thalamus, and ACC), and even the cerebellum. However, the results have not been wholly consistent. For example, some studies have reported increased activation of the insula in PTSD^27^,^29^,^30^, while others reported decreased^31^,^32^ or absent activation^24^,^33^ in the insula. There are several possible reasons for this variation. Published studies differ considerably in sample size, the demographic and clinical characteristics of the patients, differential levels in baseline activity and in the imaging protocols used. Another factor, often overlooked, is the use of different control groups: neuroimaging findings in PTSD may be compared to individuals without any history of trauma exposure (‘non-trauma controls’, NTC) or to individuals with a history of trauma exposure who have not developed PTSD (‘trauma-exposed controls’, TEC), and clearly these two comparisons have different pathophysiological implications.

Although many task-related neuroimaging meta-analyses, synthesizing a variety of symptom provocation and cognitive-emotional studies, have been performed to elucidate the neural underpinnings of PTSD^14^,^15^,^16^,^17^,^34^, discrepancies between the results for different tasks have likely contributed to the heterogeneity of the conclusions^16^,^17^,^34^. Therefore, performing a meta-analysis of resting-state neuroimaging studies which observe the brain in the absence of overt task performance or stimulation should offer the technical advantage of greatly increased homogeneity of reported studies.

In the present study, we used a voxel-based meta-analytic technique, Effect Size Signed Differential Mapping (ES-SDM), to identify consistent functional brain alterations in PTSD by integrating the full range of studies reporting resting regional brain activity. We performed two individual pooled meta-analyses, comparing PTSD with TEC and with NTC respectively, to explore the different pathophysiological implications in PTSD. In addition, we used subgroup meta-analyses to control for comorbidity and medication. Finally, we performed meta-regression analysis to examine the potential effects of age, illness severity and illness duration of PTSD patients.

## Results

### Studies included in the meta-analyses

**Figure 1:**
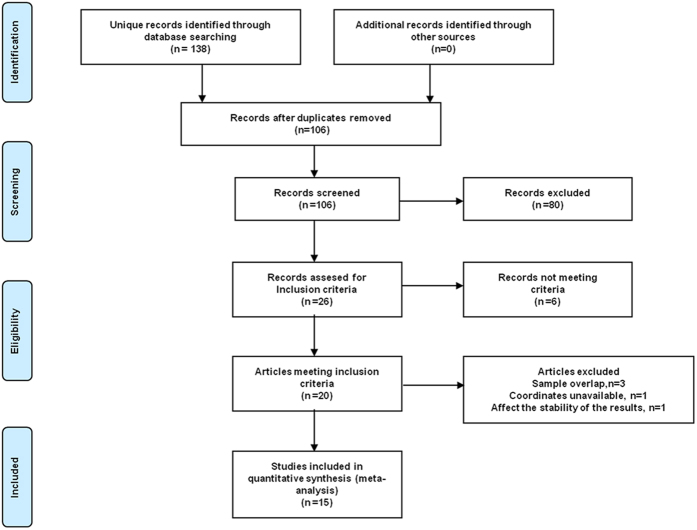
Meta-analysis of resting-state studies in PTSD.

The search strategy identified a total of 138 papers (Fig. 1), of which 19 papers^12^,^19^,^20^,^21^,^22^,^23^,^24^,^26^,^27^,^28^,^29^,^30^,^31^,^32^,^33^,^35^,^36^,^37^,^38^ met the inclusion criteria. No additional eligible articles were found in the reference lists of the selected studies. All these were in English except 4 papers^23^,^29^,^30^,^38^ in Chinese, which were translated into English for assessment. For studies that reported results for multiple analysis methods such as ALFF and ReHo with the same group or overlapping groups of participants in different publications^28^,^31^,^32^,^37^,^38^, the studies using ALFF were selected to decrease the heterogeneity of methodology (as more single-method studies used ALFF than ReHo). For 2 studies^32^,^37^ which used ALFF with overlapping groups of participants, the study with the most participants was selected. For two studies^19^,^21^ which reported between-group differences between in functional connectivity between multiple brain regions as well as in regional brain activities, we took only the regional brain activity results into account. One study^24^ used two different modalities (SPECT and PET) with the same PTSD group but different control groups, and in this case we selected the larger control group. One study^22^ which showed significant statistical heterogeneity (p < 0.005) was excluded. Finally 15 studies were included in the meta-analysis (Table 1). These included 6 studies^12^,^21^,^33^,^35^,^36^,^37^ that recruited partial PTSD patients with comorbidity and 3 studies^21^,^24^,^33^ that recruited partial PTSD patients taking medication at the time of study. Three studies^12^,^29^,^33^ employing a three-group design contributed separately to the TEC and NTC analyses. One study^21^ contributed no coordinates as no significant between-group differences in low-frequency oscillations were found.

Finally, our total sample comprised 286 patients with PTSD, 203 TECs and 155 NTCs. In the PTSD vs. TEC group were 7 studies comprising 178 PTSD and 203 TEC; controlling for comorbidity and medication there were 4 studies comprising 132 PTSD and 150 TEC, and 5 studies comprising 112 PTSD and 132 TEC. In the PTSD vs. NTC group were 11 studies comprising 149 PTSD and 155 NTC; controlling for comorbidity and medication there were 6 studies comprising 74 PTSD and 82 NTC, and 8 studies comprising 106 PTSD and 111 NTC, respectively. In no study was there any significant difference in age or sex between the PTSD and control groups.

### Meta-analysis of studies of PTSD vs. NTC

**Figure 2:**
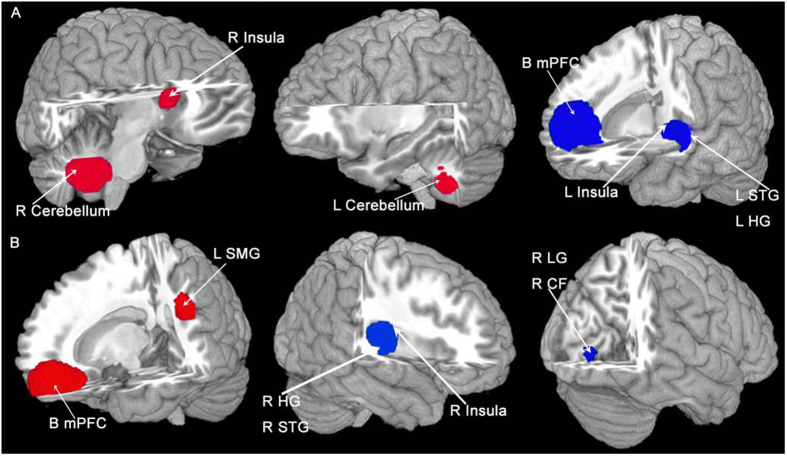
The areas of increased (red) and decreased (blue) resting-state brain activity in the meta-analyses of studies in PTSD patients compared with NTC (**A**) and TEC (**B**). R, right; L, left; (**B**), bilateral; mPFC, medial prefrontal cortex; SMG, supramarginal gyrus; STG, superior temporal gyrus; HG, Heschl’s gyrus; LG, lingual gyrus; CFC, calcarine fissure cortex; PTSD, posttraumatic stress disorder; NTC, non-traumatized controls; TEC, trauma-exposed controls.

In the pooled whole-brain meta-analysis, in PTSD compared to non-trauma controls, resting-state activity was increased in the bilateral cerebellum, right insula (anterior part) and inferior frontal gyrus (IFG), and decreased in the dorsal medial prefrontal cortex (dmPFC) (including the bilateral medial superior frontal gyrus (mSFG) and anterior cingulate gyrus (ACC; BA32)), left insula (posterior part, BA 48) and adjacent left auditory cortex (including the superior temporal gyrus (STG; BA 48) and Heschl’s gyrus (HG, BA 48)) (Fig. 2 and Table 2). The results were the same in the subgroup meta-analysis of ‘non-comorbidity’ studies compared to NTC. In the subgroup meta-analysis of ‘non-medication’ studies compared to NTC, resting-state activity in PTSD was increased in the right anterior insula, left amygdala, left parahippocampal gyrus and hippocampus, and right IFG, and decreased in the dmPFC (including the bilateral SFG and ACC).

### Meta-analysis of studies in PTSD vs. TEC

In the pooled whole-brain meta-analysis, in PTSD compared to trauma-exposed controls, resting-state activity was increased in the ventral mPFC (vmPFC, including the bilateral mSFG, bilateral gyrus rectus (BA 11) and bilateral ACC), left SMG, and middle frontal gyrus (MFG), and decreased in the right posterior insula and adjacent right auditory cortex (including the right HG and STG) and right visual cortex (including the right lingual gyrus (LG) and calcarine fissure and surrounding cortex (CFC, BA 18)) (Fig. 2 and Table 3). In the subgroup meta-analysis of non-comorbidity studies, resting-state activity in PTSD compared to TEC was increased in the vmPFC and right MFG, and decreased in the right posterior insula. The results were the same in the subgroup meta-analysis of non-medication studies compared to TEC.

### Reliability analysis

In a whole-brain jack-knife sensitivity analysis of PTSD vs. NTC (Table 4), the findings of decreased dmPFC activity in patients with PTSD were highly replicable, being preserved throughout all 11 combinations of the data sets. The results in the right cerebellum and left anterior insula were significant in all but 1 combination, and the results in the right posterior insula, right IFG, left cerebellum and left STG were significant in all but 2 combinations.

Whole-brain jack-knife sensitivity analysis of PTSD vs. TEC (Table 5) showed that the findings in bilateral vmPFC, bilateral gyrus rectus, and right posterior insula were highly replicable, being preserved in all 7 combinations of data sets. The results in left ACC, right MFG and right STG were significant in all but 1 combination, and the increased activity in left SMG and left STG was significant in all but 2 combinations.

### Meta-regression

**Figure 3:**
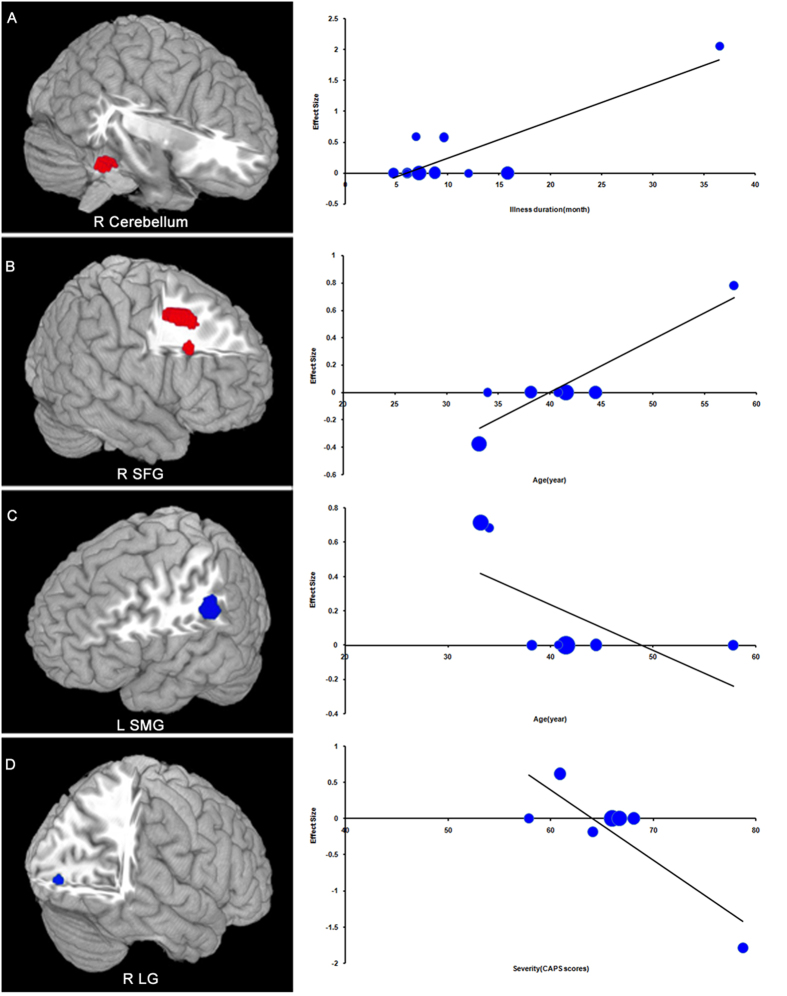
Results of meta-regression analyses of studies of PTSD patients compared with NTC (**A**) and TEC (**B–D**). (**A**) Illness duration is positively associated with the resting-state activity in the L cerebellum; (**B**) Mean patient age is positively associated with resting-state activity in the R SFG; (**C**) Mean age is negatively associated with resting-state activity in the L SMG; (**D**) Illness severity is negatively associated with resting-state activity in the R LG. Each study is represented as a dot, with larger dots symbolizing larger sample sizes. The regression line (meta-regression signed differential mapping slope) is shown as a straight line. R, right; L, left; SFG, superior frontal gyrus; SMG, supramarginal gyrus; LG, lingual gyrus; PTSD, posttraumatic stress disorder; NTC, non-traumatized controls; TEC, trauma-exposed controls.

Variables explored by regression were age and the duration and severity of illness (Fig. 3). In meta-regression analyses of studies of PTSD vs. NTC the mean illness duration was positively associated with resting-state activity in the right cerebellum; no effect of age and illness severity was detected. In meta-regression analyses of PTSD vs. TEC age was positively associated with resting-state activity in the right SFG and negatively associated with activity in the left SMG; illness severity was negatively associated with activity of right LG; no effect of illness duration was detected.

## Discussion

We used quantitative ES-SDM meta-analytic methods to synthesize findings from 15 resting-state functional neuroimaging studies of PTSD, in which patients with PTSD were compared to either TEC or NTC. The result confirmed a subset of regional differences that have frequently been reported in previous PTSD studies, including hyperactive anterior insula and hypoactive dmPFC in PTSD patients compared with NTC, and hyperactive vmPFC in PTSD compared with TEC. In addition differences were found in other regions, such as the auditory and visual cortex and cerebellum, that have hitherto been neglected in the modeling of trauma symptoms. Results were the same in subgroup meta-analysis of ‘non-comorbidity’ studies compared with NTC, and additional hyperactivity of the amygdala and hippocampus in the PTSD patients were identified in the subgroup meta-analysis of ‘non-medication’ studies compared with NTC.

### Findings in the pooled meta-analysis

In the meta-analyses, PTSD patients had increased resting-state brain activity in vmPFC compared with TEC, and decreased activity in dmPFC when compared with NTC. Alterations in mPFC have often been reported in PTSD studies using various imaging modalities including structural MRI^39^, task-related MRI^40^ and resting-state fMRI^31^. It therefore seems likely that mPFC is involved in the pathogenesis of PTSD. mPFC is a complex region, broadly divided into dorsal and ventral subdivisions, of which the dorsal part is involved in appraisal of negative emotion and detection of emotional conflict, while the ventral part has a regulatory role with respect to the limbic region in generating emotion responses^41^. MPFC is accepted as playing a critical role in cognitive and emotional dysregulation in the pathogenesis of PTSD^7^,^42^. Previous meta-analyses have often synthesized a variety of task-related studies, in which different task paradigms invoke different responses in the mPFC (such as decreased vmPFC activation in response to emotional vs. neutral scenes in PTSD patients^43^ but increased vmPFC activation during encoding of negative words^44^) that may increase the heterogeneity of the conclusions. In contrast, our results, reflecting intrinsic brain activity without the influence of external tasks, may provide more reliable information on neural patterns in mPFC and their possible roles in the pathophysiology of PTSD. However, the mPFC activity differences observed between PTSD patients and controls are not unambiguously interpretable in pathophysiological terms, and will need to be combined with results of task-related fMRI. In a self-referential cognition study, PTSD patients demonstrated less dmPFC response than did healthy controls^40^. Decreased dmPFC activity may be related to cognition in appraisal of negative emotion and resolution of conflict emotion in PTSD patients in the baseline state compared with the NTC^41^. However, increased vmPFC activity in PTSD relative to the TEC is inconsistent with the influential view that negative emotion regulation is lacking in PTSD patients owing to the hypoactive vmPFC, manifesting as failure to inhibit the hyperactive limbic regions (such as amygdala and insula)^7^. Lanius and colleagues have described a specific dissociative subtype of PTSD (defined as showing detachment from the overwhelming emotional content of the experience) that exhibits higher midline prefrontal inhibition of the limbic regions^45^. However, it is impossible to be clear about the subtype of the PTSD patients included in the present meta-analysis. Clearly the role of hyperactive dmPFC in PTSD merits further study.

We found hyperactive right anterior insula in PTSD patients compared with NTC at rest. Hyperactivity in the anterior insula has been reported in resting-state functional neuroimaging studies of PTSD^27^,^30^ as well as in multiple task-based studies^46^,^47^, which seems to be a consistent pattern across different brain states. The functions of the anterior insula include not only the generalization of interoceptive anxiety but also perception of internal states^48^. Hyperactivity in anterior insula at rest may suggest an elevated detection of and response to internal and external salient stimuli^49^. In addition, we also found hypoactivity in posterior insula and its adjacent sensory-related regions including the STG and HG in PTSD patients compared with both TEC and NTC. This is not inconsistent with the previously-mentioned hyperactivity in anterior insula, because there are well-documented structural connectivity and functional differences between the anterior and posterior insula^50^,^51^,^52^, the anterior insula being more related to self-awareness, salience detection, cognition, and other emotional/social behaviors, while the posterior part is related more to sensory perception and motor-related functions^51^,^53^. Moreover, we found hypoactivity in some sensory-related regions including the STG, HG (auditory cortex), and LG (visual cortex). Previous resting-state studies also revealed hypoactivity in the LG, cuneus^31^, and STG^54^ in PTSD. In a study investigating the resting-state network using independent component analysis, PTSD patients showed abnormal functional connectivity in the auditory and visual network, suggestive of low-level perceptual deficits^55^. Thus, hypoactivity in the posterior insula as well as these sensory-related cortices at rest may reflect decreased perception of the external environment in PTSD patients compared with both TEC and NTC. Furthermore, hypoactivity of posterior insula and its adjacent sensory-related regions was reliably present in both pooled meta-analyses of PTSD vs. TEC and PTSD vs. NTC, which may suggest that this is a true disease-related pattern. However, it is also possible that the hypoactivity in these sensory-related regions may be related to differing responses to the confining environment of the MRI scanner. Further study is warranted.

Our finding of cerebellar hyperactivity in PTSD is consistent with reports of elevated cerebellum rCBF activity in PTSD at rest^12^. Cerebellum has been implicated in the pathophysiology of PTSD, some studies reporting altered functioning^20^,^56^ and even structure^57^ of the cerebellum in PTSD. The cerebellum, traditionally associated with motor control, is increasingly implicated in cognitive processing and emotion mediation^58^,^59^, and intimate afferent and efferent connections to the prefrontal cortex provide a neuroanatomical substrate^60^,^61^. Patients with cerebellar lesions manifest a constellation of cognitive, affective and behavioral abnormalities included distractibility, disinhibition, anxiety, as well as aggression and irritability^62^. Kipping and colleagues^63^ investigating the negative affective responses to positive events in PTSD, found that negative affective interference scores positively predicted response within the right cerebellum, left amygdala, and right middle frontal gyrus. Furthermore, resting-state functional connectivity across the cerebellum has been mapped to the cerebral cortex, covering prefrontal, motor, somatosensory, posterior parietal, visual, and auditory cortices^64^. Taken together, the evidence suggests that the hyperactive cerebellum at rest may cooperate with the cerebral cortex in contributing to internal activity in PTSD.

### Findings in the subgroup meta-analysis

In the subgroup meta-analysis of non-medication studies, increased activity in the left amygdala, parahippocampal gyrus and hippocampus was observed in PTSD compared to NTC which was not identified in the pooled meta-analysis. The amygdala participates in the enhancement of startle^65^, and the hippocampus is involved in the memory retrieval^66^. Many task-based functional neuroimaging studies with non-medicated PTSD patients have shown increased activation of limbic and paralimbic structures, mainly in the amygdala, hippocampus and parahippocampus^7^,^67^. The present results might therefore suggest that activation of amygdala, hippocampus, and parahippocampus may be different in non-medicated PTSD patients. A longitudinal study in PTSD which directly investigates the relation between medication and activation of limbic structures would clearly be of interest.

### Findings in the meta-regression analyses

Meta-regression analyses of PTSD vs. NTC studies showed that the mean illness duration was positively associated with resting-state activity in the right cerebellum. This is the first study to our knowledge to report this positive relationship. Partially consistent with this, a longitudinal fMRI study found increased activation in cerebellum in acute PTSD patients and decreased cerebellar activation in symptoms-improved PTSD patients after 2 years of follow-up, which suggested the cerebellum may reflect symptom improvement^68^. Thus, we suggest that the altered cerebellar activity might accompany the development of PTSD and be some degree of restored if symptoms are improved in PTSD patients. Further studies are warranted.

We also found that PTSD symptom severity was negatively associated with activity of the right LG in the meta-regression analyses of PTSD vs. TEC studies. A previous voxel-based morphometry analysis demonstrated reduced gray matter volume in the lingual gyrus in the chronic PTSD group compared with the symptoms-improved group^39^. The result suggested a close association between the LG and PTSD symptoms which merits further study.

### Limitations

This meta-analysis has some limitations. One constraint was the availability of studies that met our criteria for inclusion. The exclusion of studies that did not report stereotaxic coordinates or used functional connectivity approaches likely reduced our power to detect less-robust activations. Further, the small number of studies precluded separate meta-analyses for some moderator variables, such as the characteristics of patients (trauma type, gender), imaging method (fMRI, PET, SPECT), and analysis method (ALFF, ReHo, rCBF, rCMRglu). Although we conducted subgroup meta-analyses of ‘non-comorbidity’ and ‘non-medication’ studies in PTSD compared with TEC, these included only 4 studies and 5 studies, respectively, and had limited power; further investigation will needed to determine their relative contributions to PTSD pathology.

In addition, like all coordinate-based methods, ES-SDM assumes that effect sizes originate from homogeneous t-value contrasts; in fact they might originate from different covariate models or from different raw statistics, and this limitation could be controlled by SDM covariate analyses, if relevant. Finally, all neuroimaging data are highly sensitive to common artifacts such as head motion and breathing effects that may influence the results^69^,^70^.

## Conclusions

The present meta-analyses provide a unique opportunity to assess altered intrinsic brain activities across individual PTSD studies during rest. The results confirmed a subset of consistent regional differences often reported in previous PTSD studies, including the vmPFC, insula, and limbic regions (including the amygdala and hippocampus). Additional regions were found, such as the auditory and visual cortex and cerebellum, that have received less attention. It is noteworthy that different parts of mPFC and insula may have different pathophysiological implications in PTSD, and future PTSD studies should subdivide these regions, especially in functional connectivity analysis. Differential brain regions and activities found in two individual pooled meta-analyses revealed differential pathophysiological implications in two different comparisons. Further studies are needed to determine whether the findings reported here are disease-related or stress-related. Subgroup analyses also suggested an influence of medication and co-morbidity on the pathophysiology of PTSD, which will need to be verified by further study.

Finally, it must be acknowledged that differences observed between PTSD and controls during resting-state fMRI are still not easily interpreted, because diverse interpersonal differences (e.g. drugs, smoking, mental state, and many other confounders) may influence the neuroimaging result. Transcending this limitation will require innovative methodological approaches. This is a developing field and our results should be considered provisional.

## Methods

### Study selection

A systematic search strategy was used to select studies published between January 1995 and April 2015. A combination search strategy of Mesh terms and text words was conducted in PubMed, Web of Knowledge, Embase and Science Direct, China National Knowledge Infrastructure (CNKI), National Technical Information Service, and System for Information on Grey Literature. The terms used were as follows: ALFF <or> ReHo <or> rCBF <or> rCMRglu <or> ASL <or> amplitude of low frequency fluctuations <or> low frequency fluctuations <or> regional homogeneity <or> regional cerebral perfusion <or> regional cerebral metabolic <or> arterial spin labeling; PTSD <or> posttraumatic stress disorder; baseline <or> resting-state <or> rest <or> resting. We assessed all search results for potential suitability. The abstracts were all in English; articles in Chinese were translated into English for assessment. Studies were selected according to the following inclusion criteria: 1) the study had used at least one of the functional imaging techniques of fMRI, PET, or SPECT to analyze altered spontaneous brain activity in patients with PTSD; 2) the study included comparison of PTSD patients with NTC or TEC; 3) 3-dimensional coordinates in stereotactic space of the activation areas were clearly reported. Studies were excluded if they were case reports, reported only region of interest (ROI) findings or used seed-voxel-based analysis procedures, if the participants were not classified using current diagnostic criteria for PTSD, if the data contributed to another publication (in which case the publication with the largest group size was selected), or if the data, when added in, tipped the balance into significant heterogeneity (p < 0.005). The reference lists of the identified articles were searched for additional studies. Two authors (T.W and J.L) independently conducted the literature search. The results were compared, any inconsistent result was discussed, and a consensus decision was reached.

### Study quality assessment

Individual study quality was assessed using a 10-point checklist, which focused on the clinical and demographic aspects of the study samples and the imaging methodology (see supplementary material, Table S1). The checklist was based on previous meta-analytic studies^71^,^72^. The assessment included the quality of the diagnostic procedures, the demographic and clinical characterization, the sample size, the MRI acquisition parameters, the analysis method and the quality of the reported results. Though the checklist was not designed as an assessment tool, it provides some objective indication of the rigor of individual studies. At least two authors reviewed every paper and independently determined a quality rating. These ratings were compared, any disagreement was discussed, and a consensus score was obtained. The study quality scores are presented in Table 1.

### Voxel-wise meta-analysis

Papers were divided into two based upon the nature of the control group, TEC or NTC (i.e. with and without trauma exposure, as defined in the Introduction). Two individual meta-analyses were performed comparing PTSD with TEC and with NTC. Additional subgroup meta-analyses were performed to control for comorbidity and medication. The meta-analyses were performed using ES-SDM (Effect Size Signed Differential Mapping; http://www.sdmproject.com/software)^73^,^74^,^75^ which uses peak coordinates to recreate, for each study, a map of the effect sizes of the differences between patients and controls, and then conducts a standard random-effects variance-weighted meta-analysis in each voxel^75^. Specifically, first peak coordinates and effect-sizes (e.g. t-values or z-scores) of all functional differences that were statistically significant at the whole-brain level between patients and controls were extracted from each dataset. We checked that each included study used the same statistical threshold throughout the whole brain, to avoid potential bias toward liberally-thresholded regions. For studies that reported only z-scores, these were converted to t-values using the online converter (www.sdmproject.com/utilities/?show=Statistics). For studies not reporting any measure related to effect size (t-values, z-scores, p-values or similar), we write a “p” for positive peaks (i.e. patients > controls) and an “n” for negative peaks (i.e. patients < controls), according to the SDM tutorials. Second, peak coordinates and their t-values were used to recreate a standard Montreal Neurological Institute (MNI) map of the differences for each study by means of a non-normalized Gaussian kernel, which assigns higher effect sizes to the voxels more correlated with peaks. In the assignment, a relatively large full-width at half-maximum (FWHM, 20 mm) was used to control false positive results^74^. Unlike earlier meta-analytic methods such as activation likelihood estimation^76^ and multilevel kernel density analysis^77^, both positive and negative coordinates are reconstructed in the same map to avoid any voxel erroneously appearing to be positive and negative at the same time^73^. Third, the mean map was obtained by voxel-wise calculation of the random-effects mean of the study maps, weighted by the sample size and variance of each study and the between-study heterogeneity. Finally, statistical significance was calculated using standard randomization tests^75^, creating null distributions from which the p-values were obtained directly. Default ES-SDM kernel size and thresholds were used (FWHM = 20 mm, voxel p = 0.005, peak height Z = 1, cluster extent = 10 voxels)^74^.

### Reliability analysis

Systematic whole-brain voxel-based jack-knife sensitivity analysis was conducted to test the robustness of the results of the two main meta-analyses, PTSD vs. NTC and PTSD vs. TEC. Briefly, jack-knife sensitivity analysis consists of repeating the analysis discarding just one study each time, and is used to assess the reproducibility of the results across different studies^74^. The rationale is that if a previously significant brain region remains significant in all or most of the combinations of studies, it can be concluded that this finding is highly replicable^78^.

### Meta-regression analysis

The potential effects of age, illness severity and illness duration of PTSD patients were examined by simple linear regression, weighted by the square root of the sample size and restricted to predict only possible SDM values (i.e. from −1 to 1) in the observed range of values of the variable. The main output for each variable was a map of the regression slope^78^. As in previous meta-analyses, to minimize the detection of spurious associations we decreased the probability threshold to 0.0005, required abnormalities to be detected both in the slope and in one of the extremes of the regressor, and discarded findings in regions other than those detected in the main analyses. Finally, regression plots were visually inspected to discard fits driven by too few studies^78^.

## Additional Information

**How to cite this article**: Wang, T. *et al.* Altered resting-state functional activity in posttraumatic stress disorder: A quantitative meta-analysis. *Sci. Rep.* **6**, 27131; doi: 10.1038/srep27131 (2016).

## Supplementary Material

Supplementary Information

## Figures and Tables

**Table 1:** Summary of studies included in the meta-analysis.

Study	Modality/Analysis	Trauma type	Group; no.(female)			Group; mean age(y)			MID	CAPS	DS	Thre	Co	QS
			PTSD	TEC	HC	PTSD	TEC	HC						
Baojuan *et al.*^19^	ASL-fMRI/rCBF	Mine disaster	10(0)	10(0)	–	41	34	–	6	79	Drug-naïve	uncorr	N	10
Bing *et al.*^20^	rs-fMRI/ALFF	MVC	20(7)	–	20(6)	33	–	3	7	52	Drug-free	corr	N	10
Bluhm *et al.*^21^	rs-fMRI/ALFF	SA	17(17)	–	15(15)	39	–	38	>6	77	Drug	corr	Y	9
Bonne *et al.*^12^	SPECT/rCBF	Mixed	11(7)	17(9)	11(6)	34	35	33	7	58	Drug-naïve	uncorr	Y	9
Huang *et al.*^23^	rs-fMRI/ALFF	Mixed	10(7)	–	10(7)	33	–	32	16	NA	Drug-naïve	uncorr	N	9
Kim *et al.*^35^	SPECT/rCBF	Subway fire	19(13)	–	19(7)	27	–	32	15	71	Drug-free	corr	Y	9.5
Kim *et al.*^24^	PET/rCMRglu	SA	12(12)	–	15(15)	36	–	38	10	NA	Drug	uncorr	N	9
Semple *et al.*^26^	PET/rCBF	Combat	7(0)	–	6(0)	43	–	34	>12	NA	Drug-free	uncorr	N	8.5
Shin *et al.*^33^	PET/rCMRglu	Combat	14(0)	19(0)	14(0)	58	57	58	>6	66	Drug	uncorr	Y	9
Song *et al.*^29^	rs-fMRI/ALFF	Burn	16(1)	16(1)	16(1)	38	36	39	9	68	Drug-free	corr	N	10
Yan *et al.*^27^	rs-fMRI/ALFF	Combat	52(0)	52(0)	–	33	34	–	>6	67	NA	corr	N	9.5
Yin *et al.*^31^	rs-fMRI/ALFF	Earthquake	54(39)	72(50)	–	42	42	–	8	64	Drug-naïve	corr	N	10
Zhang *et al.*^30^	rs-fMRI/ReHo	MVC	9(4)	–	15(7)	33	–	26	>6	NA	Drug-naïve	uncorr	N	9
Zhong *et al.*^36^	rs-fMRI/ReHo	Mixed	14(8)	–	14(8)	31	–	29	5	68	Drug-naïve	corr	Y	9
Zhu *et al.*^37^	rs-fMRI/ALFF	Earthquake	21(17)	17(12)	–	47	43	–	48	69	Drug-naïve	corr	Y	9.5

TEC, trauma-exposed controls without PTSD; NTC, non-traumatized controls without PTSD; SA, sexual abuse/assault; MVA, motor vehicle accident; SPECT, single-photon emission computed tomography; PET, positron emission tomography; rs-fRMI, resting-state functional magnetic resonance imaging; ASL, arterial spin labeling; rCBF, regional cerebral blood flow; ALFF, amplitude of low-frequency fluctuation; ReHo, regional homogeneity; rCMRglu, regional cerebral glucose metabolic rate; CAPS, clinician-administered PTSD scale; MID, Mean illness duration (months); DS, Drug state; Thre, Threshold; Co, Comorbidity; QS, Quality score (out of 10); NA, not available; Y, yes; N, no.

**Table 2:** Brain regions showing greater and less activity in PTSD vs. NTC (voxelwise uncorrected p < 0.005 and FWHM 20 mm).

Brain Regions	Maximum			Clusters	
	MNI coordinates, x, y, z	SDM value	p-value	No. voxel	Breakdown (no. of voxels)
**Pooled meta-analysis**					
*PTSD* > *NTC*					
R cerebellum	30, −38, −40	1.808	0.000372	714	R cerebellum, hemispheric lobule X (608)
					M cerebellar peduncle (106)
R insula, BA 48	32, 16, 2	1.563	0.001783	101	R insula, BA 48 (90)
					R external capsule (6)
					R lenticular nucleus, putamen, BA 48 (5)
L cerebellum	−32, −46, −46	1.449	0.003311	190	L cerebellum, hemispheric lobule VIII (183)
					M cerebellar peduncle (7)
R inferior frontal gyrus, BA 44	50, 12, 18	1.432	0.003644	40	R inferior frontal gyrus, opercular part, BA 44 (40)
*PTSD* < *NTC*					
R superior frontal gyrus, medial, BA 10	6, 50, 6	−1.327	0.000541	1496	R superior frontal gyrus, medial, BA 10 (374)
					L superior frontal gyrus, medial, BA 10 (502)
					R anterior cingulate/paracingulate gyri, BA 32 (402)
					L anterior cingulate/paracingulate gyri, BA 32 (216)
					R cingulum (cingulate gyrus) (2)
L rolandic operculum, BA 48	−44, −10, 6	−1.129	0.001809	411	L insula, BA 48 (89)
					L superior temporal gyrus, BA 48 (140)
					L rolandic operculum, BA 48 (106)
					L Heschl’s gyrus, BA 48 (76)
**Subgroup meta-analysis of studies without comorbidity**					
*PTSD* > NTC					
R cerebellum	12, −92, −26	1.597	0.001193	204	R cerebellum, crus I (204)
R insula, BA 48	36, 20, 12	1.55	0.001577	384	R insula, BA 48 (175)
					R inferior frontal gyrus, BA 48 (195)
					R lenticular nucleus, putamen, BA 48 (9)
					R external capsule (3)
					R rolandic operculum, BA 48 (2)
R cerebellum	30, −36, −42	1.527	0.001779	237	R cerebellum (237)
*PTSD* < *NTC*					
R superior frontal gyrus, medial, BA 10	6, 62, 6	−1.528	0.000382	1443	R superior frontal gyrus, medial, BA 10 (457)
					L superior frontal gyrus, medial, BA 10 (674)
					R anterior cingulate/paracingulate gyri, BA 32 (246)
					L anterior cingulate/paracingulate gyri, BA 32 (60)
					R median cingulate/paracingulate gyri, BA 32 (5)
					R cingulum(cingulate gyrus) (1)
L insula, BA 48	−38, −6, 6	−1.432	0.000992	873	L insula, BA 48 (281)
					L superior temporal gyrus, BA 48 (248)
					L rolandic operculum, BA 48 (211)
					L Heschl’s gyrus, BA 48 (107)
					L External capsule (19)
					L postcentral gyrus, BA 48 (7)
**Subgroup meta-analysis of studies without medication**					
PTSD > *NTC*					
R insula, BA 48	32, 14, 2	1.764	0.000693	228	R insula, BA 48 (146)
					R external capsule (50)
					R lenticular nucleus, putamen, BA 48 (18)
					R inferior frontal gyrus, BA 48 (14)
L amygdala, BA 34	−28, 0, −26	1.727	0.000865	333	L amygdala, BA 34 (115)
					L parahippocampal gyrus, BA 28 (83)
					L superior temporal gyrus, BA 38 (72)
					L uncinate fasciculus (19)
					L hippocampus, BA 36 (42)
					L fusiform gyrus, BA 36 (1)
					L fornix (cres)/Striaterminalis (1)
R inferior frontal gyrus, BA 44	52, 12, 18	1.602	0.001822	125	R inferior frontal gyrus, BA 44 (123)
					R precentral gyrus, BA 44 (1)
					R rolandic operculum, BA 48 (1)
PTSD < NTC					
R superior frontal gyrus, medial, BA 10	4, 40, 12	−1.459	0.000206	1828	R superior frontal gyrus, medial, BA 10 (492)
					L superior frontal gyrus, medial, BA 10 (637)
					R anterior cingulate/paracingulate gyri, BA 32 (463)
					L anterior cingulate/paracingulate gyri, BA 32 (234)
					R cingulum(cingulate gyrus) (2)
R thalamus	10, −28, 16	−1.147	0.001558	18	R thalamus (16)
					R hippocampus (2)
Genu_of_corpus_callosum	10, 22, −2	−1.098	0.002076	9	Genu of corpus callosum (7)
					R caudate nucleus, BA 11 (2)

L, Left; R, right; BA, Brodmann area; MNI, Montreal Neurological Institute; M, Middle; SDM, signed differential mapping.

**Table 3:** Brain regions showing greater and less activity in PTSD vs. TEC (voxelwise uncorrected p < 0.005 and FWHM 20 mm).

Brain Regions	Maximum			Cluster	
	MNI coordinates x, y, z	SDM value	p-value	No. of voxel	Breakdown (no. of voxels)
**Pooled meta-analysis**					
*PTSD* > *TEC*					
L superior frontal gyrus, medial orbital, BA 11	−2, 48, −10	2.915	1.45E-05	1407	R superior frontal gyrus, medial orbital, BA 11 (448)
					L superior frontal gyrus, medial orbital, BA 11 (431)
					L gyrus rectus, BA 11 (214)
					R gyrus rectus, BA 11 (180)
					L anterior cingulate/paracingulate gyri, BA 10 (118)
					R anterior cingulate//paracingulate gyri, BA 11 (16)
L supramarginal gyrus, BA 48	−54, −28, 32	1.745	0.001651	169	L supramarginal gyrus, BA 48 (117)
					L superior temporal gyrus, BA 42 (51)
					L rolandic operculum, BA 48 (1)
R middle frontal gyrus, BA 46	40, 42, 36	1.676	0.002254	59	R middle frontal gyrus, BA 46 (59)
*PTSD* < *TEC*					
R Heschl’s gyrus, BA 48	38, −22, 8	−1.681	0.000941	370	R insula, BA 48 (141)
					R rolandic operculum, BA 48 (104)
					R Heschl’s gyrus, BA 48 (85)
					External_capsule_R (29)
					R superior temporal gyrus, BA 48 (11)
R calcarine fissure/surrounding cortex, BA 18	14, −78, 2	−1.874	0.000476	362	R calcarine fissure/surrounding cortex, BA 17 (259)
					R lingual gyrus, BA 18 (67)
					R cuneus cortex, BA 17 (34)
					L calcarine fissure/surrounding cortex (1)
					R superior occipital gyrus, BA 18 (1)
R fusiform gyrus, BA 37	38, −56, −14	−1.69	0.000904	88	R fusiform gyrus, BA 37 (83)
					R cerebellum, BA 37 (5)
R caudate nucleus	8, 6, 8	−1.48	0.004387	5	R caudate nucleus (5)
**Subgroup meta-analysis of studies without comorbidity**					
PTSD > *TEC*					
L superior frontal gyrus, medial orbital, BA 11	−2, 48, −12	3.425	9.29E-06	2012	R superior frontal gyrus, medial orbital, BA 11 (612)
					L superior frontal gyrus, medial orbital, BA 11 (545)
					L gyrus rectus, BA 11 (347)
					R gyrus rectus, BA 11 (242)
					L anterior cingulate/paracingulate gyri, BA 10 (210)
					R anterior cingulate/paracingulate gyri, BA 11 (52)
					L anterior corona radiata (3)
					L olfactory cortex (1)
R middle frontal gyrus, BA 46	42, 42, 34	1.522	0.00243	100	R middle frontal gyrus, BA 46 (100)
PTSD < TEC					
R fusiform gyrus, BA 37	42, −58, −18	−1.743	0.000846	307	R cerebellum, BA 37 (154)
					R fusiform gyrus, BA 37 (151)
					R inferior temporal gyrus, BA 37 (2)
R calcarine fissure/surrounding cortex, BA 18	16, −98, 0	−1.739	0.00086	542	R calcarine fissure/surrounding cortex, BA 17 (338)
					R lingual gyrus, BA 18 (104)
					R cuneus cortex, BA 17 (68)
					L calcarine fissure/surrounding cortex (22)
					R superior occipital gyrus, BA 18 (9)
					L cuneus cortex, BA 18 (1)
R rolandic operculum, BA 48	50, −12, 12	−1.524	0.00188	207	R rolandic operculum, BA 48 (68)
					R insula, BA 48 (54)
					R Heschl’s gyrus, BA 48 (30)
					R external capsule (27)
					R superior temporal gyrus, BA 48 (25)
					R lenticular nucleus, putamen, BA 48 (3)
**Subgroup meta-analysis of studies without medication**					
PTSD > *TEC*					
R gyrus rectus, BA 11	4, 46, −18	2.026	0.000549	648	R superior frontal gyrus, medial orbital, BA 11 (322)
					L superior frontal gyrus, medial orbital, BA 11 (156)
					R gyrus rectus, BA 11 (95)
					L gyrus rectus, BA 11 (53)
					L anterior cingulate/paracingulate gyri, BA 10 (22)
R middle frontal gyrus, BA 46	40, 40, 30	2.024	0.000569	147	R middle frontal gyrus, BA 46 (147)
PTSD < *TEC*					
R Heschl’s gyrus, BA 48	44, −16, 10	−2.024	0.000249	381	R insula, BA 48 (133)
					R rolandic operculum, BA 48 (115)
					R Heschl’s gyrus, 0.000249 (76)
					R external capsule (36)
					R superior temporal gyrus, BA 48 (21)
R calcarine fissure/surrounding cortex, BA 18	12, −92, 8	−2.389	1.03E-05	405	R calcarine fissure/surrounding cortex, BA 18 (270)
					R lingual gyrus, BA 18 (76)
					R cuneus cortex (53)
					R superior occipital gyrus, BA 18 (3)
					L calcarine fissure/surrounding cortex, BA 17 (3)
R fusiform gyrus, BA 37	38, −56, −14	−2.123	0.000135	145	R fusiform gyrus, BA 37 (125)
					R lingual gyrus, BA 19 (16)
					R cerebellum, BA 37 (3)
					R inferior temporal gyrus, BA 37 (1)

L, Left; R, right; BA, Brodmann area; MNI, Montreal Neurological Institute; SDM, signed differential mapping.

**Table 4:** Sensitivity analyses of studies in the meta-analysis of PTSD vs. NTC.

Discarded studies	Hyperactivation regions				Hypoactivation regions					
	R cerebellum	R insula	R IFG	L cerebellum	R mSFG	L mSFG	L ACC	R ACC	L insula	L STG
Bing *et al.*^20^	Y	Y	Y	Y	Y	Y	Y	Y	Y	Y
Bluhm *et al.*^21^	Y	Y	Y	Y	Y	Y	Y	Y	Y	Y
Bonne *et al.*^12^	Y	Y	Y	N	Y	Y	Y	Y	Y	Y
Huang *et al.*^23^	Y	Y	Y	Y	Y	Y	Y	Y	N	N
Kim *et al.*^35^	Y	Y	Y	Y	Y	Y	Y	Y	Y	Y
Kim *et al.*^24^	N	Y	Y	N	Y	Y	Y	Y	N	N
Semple *et al.*^26^	Y	N	N	Y	Y	Y	Y	Y	Y	Y
Shin *et al.*^33^	Y	Y	Y	Y	Y	Y	Y	Y	Y	Y
Song *et al.*^29^	Y	N	N	Y	Y	N	N	Y	Y	Y
Zhang *et al.*^30^	Y	Y	Y	Y	Y	Y	Y	Y	Y	Y
Zhong *et al.*^36^	Y	Y	Y	Y	Y	Y	Y	Y	Y	Y

L, left; R, right; IFG, inferior frontal gyrus; mSFG, medial superior frontal gyrus; ACC, anterior cingulate gyri; STG, superior temporal gyrus; Y, yes.

**Table 5:** Sensitivity analyses of studies in the meta-analysis of PTSD vs. TEC.

Discarded studies	Hyperactivation regions								Hypoactivation regions	
	L mSFG	R mSFG	L GR	R GR	L ACC	L SMG	L STG	R MFG	R insula	R STG
Baojuan *et al.*^19^	Y	Y	Y	Y	Y	Y	Y	Y	Y	Y
Bonne *et al.*^12^	Y	Y	Y	Y	Y	N	N	Y	Y	Y
Shin *et al.*^33^	Y	Y	Y	Y	Y	Y	Y	Y	Y	Y
Song *et al.*^29^	Y	Y	Y	Y	Y	Y	Y	Y	Y	Y
Yan *et al.*^27^	Y	Y	Y	Y	Y	N	N	Y	Y	Y
Yin *et al.*^31^	Y	Y	Y	Y	N	Y	Y	N	Y	N
Zhu *et al.*^37^	Y	Y	Y	Y	Y	Y	Y	Y	Y	Y

L, left; R, right; mSFG, medial superior frontal gyrus; GR, gyrus rectus; SMG, supramarginal gyrus; ACC, anterior cingulate gyri; STG, superior temporal gyrus; MFG, middle frontal gyrus; Y, yes; N, no.

## References

[b1] Association A. P. Diagnostic and Statistical Manual of Mental Disorders 5th edition. American Psychiatric Association (2013).

[b2] Hayes J. P., Vanelzakker M. B. & Shin L. M. Emotion and cognition interactions in PTSD: a review of neurocognitive and neuroimaging studies. Frontiers in integrative neuroscience 6, 89, doi: 10.3389/fnint.2012.00089 (2012). 23087624 PMC3466464

[b3] Lanius R. A., Bluhm R., Lanius U. & Pain C. A review of neuroimaging studies in PTSD: Heterogeneity of response to symptom provocation. Journal of Psychiatric Research 40, 709–729, doi: 10.1016/j.jpsychires.2005.07.007 (2006). 16214172

[b4] Francati V., Vermetten E. & Bremner J. D. Functional neuroimaging studies in posttraumatic stress disorder: review of current methods and findings. Depression and anxiety 24, 202–218, doi: 10.1002/da.20208 (2007). 16960853 PMC3233765

[b5] Mahan A. L. & Ressler K. J. Fear conditioning, synaptic plasticity and the amygdala: implications for posttraumatic stress disorder. Trends Neurosci 35, 24–35, doi: 10.1016/j.tins.2011.06.007 (2012). 21798604 PMC3206195

[b6] Nemeroff C. B. *et al.* Posttraumatic stress disorder: a state-of-the-science review. J Psychiatr Res 40, 1–21, doi: 10.1016/j.jpsychires.2005.07.005 (2006). 16242154

[b7] Hughes K. C. & Shin L. M. Functional neuroimaging studies of post-traumatic stress disorder. Expert review of neurotherapeutics 11, 275–285, doi: 10.1586/ern.10.198 (2011). 21306214 PMC3142267

[b8] Biswal B., Yetkin F. Z., Haughton V. M. & Hyde J. S. Functional Connectivity in the Motor Cortex of Resting Human Brain Using Echo-Planar Mri. Magnet Reson Med 34, 537–541, doi: 10.1002/mrm.1910340409 (1995). 8524021

[b9] Zang Y. F., Jiang T. Z., Lu Y. L., He Y. & Tian L. X. Regional homogeneity approach to fMRI data analysis. Neuroimage 22, 394–400, doi: 10.1016/j.neuroimage.2003.12.030 (2004). 15110032

[b10] Jueptner M. & Weiller C. Review - Does Measurement of Regional Cerebral Blood-Flow Reflect Synaptic Activity - Implications for Pet and Fmri. Neuroimage 2, 148–156, doi: 10.1006/nimg.1995.1017 (1995). 9343597

[b11] Zang Y. F. *et al.* Altered baseline brain activity in children with ADHD revealed by resting-state functional MRI. Brain & development 29, 83–91, doi: 10.1016/j.braindev.2006.07.002 (2007). 16919409

[b12] Bonne O. *et al.* Resting regional cerebral perfusion in recent posttraumatic stress disorder. Biological psychiatry 54, 1077–1086 (2003). 14625150 10.1016/s0006-3223(03)00525-0

[b13] Ishii K., Ikerjiri Y., Sasaki M., Kitagaki H. & Mori E. Regional cerebral glucose metabolism and blood flow in a patient with Marchiafava-Bignami disease. AJNR. American journal of neuroradiology 20, 1249–1251 (1999). 10472981 PMC7055969

[b14] Etkin A. & Wager T. D. Functional neuroimaging of anxiety: a meta-analysis of emotional processing in PTSD, social anxiety disorder, and specific phobia. The American journal of psychiatry 164, 1476–1488, doi: 10.1176/appi.ajp.2007.07030504 (2007). 17898336 PMC3318959

[b15] Hayes J. P., Hayes S. M. & Mikedis A. M. Quantitative meta-analysis of neural activity in posttraumatic stress disorder. Biology of mood & anxiety disorders 2, 9, doi: 10.1186/2045-5380-2-9 (2012). 22738125 PMC3430553

[b16] Patel R., Spreng R. N., Shin L. M. & Girard T. A. Neurocircuitry models of posttraumatic stress disorder and beyond: A meta-analysis of functional neuroimaging studies. Neuroscience & Biobehavioral Reviews 36, 2130–2142, doi: 10.1016/j.neubiorev.2012.06.003 (2012). 22766141

[b17] Ramage A. E. *et al.* A coordinate-based meta-analytic model of trauma processing in posttraumatic stress disorder. Human brain mapping 34, 3392–3399, doi: 10.1002/hbm.22155 (2013). 22936519 PMC3514575

[b18] Shehzad Z. *et al.* The resting brain: unconstrained yet reliable. Cerebral cortex (New York, N.Y.: 1991) 19, 2209–2229, doi: 10.1093/cercor/bhn256 (2009). PMC3896030 19221144

[b19] Baojuan L., Jian L., Yang L., Hongbing L. & Hong Y. Altered resting-state functional connectivity in post-traumatic stress disorder: a perfusion MRI study. Proceedings of the SPIE - The International Society for Optical Engineering 8673, 867318, doi: 10.1117/12.2008168 (2013).

[b20] Bing X. *et al.* Alterations in the cortical thickness and the amplitude of low-frequency fluctuation in patients with post-traumatic stress disorder. Brain research 1490, 225–232, doi: 10.1016/j.brainres.2012.10.048 (2013). 23122880

[b21] Bluhm R. L. *et al.* Alterations in default network connectivity in posttraumatic stress disorder related to early-life trauma. Journal of psychiatry & neuroscience : JPN 34, 187–194 (2009). 19448848 PMC2674971

[b22] Chung Y. A. *et al.* Alterations in cerebral perfusion in posttraumatic stress disorder patients without re-exposure to accident-related stimuli. Clinical neurophysiology : official journal of the International Federation of Clinical Neurophysiology 117, 637–642, doi: 10.1016/j.clinph.2005.10.020 (2006). 16426890

[b23] Huang Q. *et al.* A fMRI study of the amygdala with posttraumatic stress disorder under the resting-state. Chin J Nerv Ment Dis 37, 705–709 (2011).

[b24] Kim S. Y. *et al.* Resting cerebral glucose metabolism and perfusion patterns in women with posttraumatic stress disorder related to sexual assault. Psychiatry research 201, 214–217, doi: 10.1016/j.pscychresns.2011.08.007 (2012). 22464826

[b25] Lanius R. A. *et al.* Default mode network connectivity as a predictor of post-traumatic stress disorder symptom severity in acutely traumatized subjects. Acta psychiatrica Scandinavica 121, 33–40, doi: 10.1111/j.1600-0447.2009.01391.x (2010). 19426163

[b26] Semple W. E. *et al.* Higher brain blood flow at amygdala and lower frontal cortex blood flow in PTSD patients with comorbid cocaine and alcohol abuse compared with normals. Psychiatry 63, 65–74 (2000). 10855761 10.1080/00332747.2000.11024895

[b27] Yan X. *et al.* Spontaneous brain activity in combat related PTSD. Neuroscience letters 547, 1–5, doi: 10.1016/j.neulet.2013.04.032 (2013). 23643995

[b28] Yin Y. *et al.* Altered regional homogeneity in post-traumatic stress disorder: a resting-state functional magnetic resonance imaging study. Neuroscience bulletin 28, 541–549, doi: 10.1007/s12264-012-1261-3 (2012). 22961475 PMC5561913

[b29] Song L. *et al.* Resting-state fMRI observation on changes of amplitude of low-frequency fluctuation in burn patients with posttraumatic stress disorder. Chinese Journal of medical imaging Technology 30, 204–208 (2014).

[b30] Zhang Y. *et al.* Regional homogeneity of resting-state brain activity in patients with posttraumatic stress disorder. J Third Mil Med Univ 34, 2260–2263 (2012).

[b31] Yin Y. *et al.* Abnormal baseline brain activity in posttraumatic stress disorder: a resting-state functional magnetic resonance imaging study. Neuroscience letters 498, 185–189, doi: 10.1016/j.neulet.2011.02.069 (2011). 21376785

[b32] Zhu H. *et al.* Altered spontaneous neuronal activity of visual cortex and medial anterior cingulate cortex in treatment-naive posttraumatic stress disorder. Comprehensive psychiatry 55, 1688–1695, doi: 10.1016/j.comppsych.2014.06.009 (2014). 25060989

[b33] Shin L. M. *et al.* Resting metabolic activity in the cingulate cortex and vulnerability to posttraumatic stress disorder. Archives of general psychiatry 66, 1099–1107, doi: 10.1001/archgenpsychiatry.2009.138 (2009). 19805700 PMC3752096

[b34] Sartory G. *et al.* In search of the trauma memory: a meta-analysis of functional neuroimaging studies of symptom provocation in posttraumatic stress disorder (PTSD). Plos One 8, e58150, doi: 10.1371/journal.pone.0058150 (2013). 23536785 PMC3607590

[b35] Kim S. J. *et al.* Decreased cerebral blood flow of thalamus in PTSD patients as a strategy to reduce re-experience symptoms. Acta psychiatrica Scandinavica 116, 145–153, doi: 10.1111/j.1600-0447.2006.00952.x (2007). 17650277

[b36] Zhong Y. *et al.* Altered cortical and subcortical local coherence in PTSD: evidence from resting-state fMRI. Acta radiologica (Stockholm, Sweden: 1987), doi: 10.1177/0284185114537927 (2014). 24973255

[b37] Zhu H. *et al.* Altered spontaneous neuronal activity in chronic posttraumatic stress disorder patients before and after a 12-week paroxetine treatment. Journal of affective disorders 174, 257–264, doi: 10.1016/j.jad.2014.11.053 (2015). 25527996

[b38] Zhu H. *et al.* A Study of Resting State Functional Magnetic Resonance Imaging in Patients with Posttraumatic Stress Disorder Using Regional Homogeneity. West China Medical Journal 27, 641–646 (2012).

[b39] Tan L. *et al.* Brain structure in post-traumatic stress disorder: A voxel-based morphometry analysis. Neural regeneration research 8, 2405–2414, doi: 10.3969/j.issn.1673-5374.2013.26.001 (2013). 25206550 PMC4146106

[b40] Bluhm R. L. *et al.* Neural correlates of self-reflection in post-traumatic stress disorder. Acta psychiatrica Scandinavica 125, 238–246, doi: 10.1111/j.1600-0447.2011.01773.x (2012). 22007877

[b41] Etkin A., Egner T. & Kalisch R. Emotional processing in anterior cingulate and medial prefrontal cortex. Trends in Cognitive Sciences 15, 85–93, doi: 10.1016/j.tics.2010.11.004 (2011). 21167765 PMC3035157

[b42] Brown V. M. & Morey R. A. Neural Systems for Cognitive and Emotional Processing in Posttraumatic Stress Disorder. Frontiers in Psychology 3, 449, doi: 10.3389/fpsyg.2012.00449 (2012). 23162499 PMC3498869

[b43] Moser D. A. *et al.* Violence-related PTSD and neural activation when seeing emotionally charged male-female interactions. Social cognitive and affective neuroscience 10, 645–653, doi: 10.1093/scan/nsu099 (2015). 25062841 PMC4420740

[b44] Thomaes K. *et al.* Increased anterior cingulate cortex and hippocampus activation in Complex PTSD during encoding of negative words. Social cognitive and affective neuroscience 8, 190–200, doi: 10.1093/scan/nsr084 (2013). 22156722 PMC3575721

[b45] Lanius R. A. *et al.* Emotion modulation in PTSD: Clinical and neurobiological evidence for a dissociative subtype. The American journal of psychiatry 167, 640–647, doi: 10.1176/appi.ajp.2009.09081168 (2010). 20360318 PMC3226703

[b46] Fonzo G. A. *et al.* Exaggerated and disconnected insular-amygdalar blood oxygenation level-dependent response to threat-related emotional faces in women with intimate-partner violence posttraumatic stress disorder. Biological psychiatry 68, 433–441, doi: 10.1016/j.biopsych.2010.04.028 (2010). 20573339 PMC2921473

[b47] Bruce S. E. *et al.* Altered emotional interference processing in the amygdala and insula in women with Post-Traumatic Stress Disorder. NeuroImage. Clinical 2, 43–49, doi: 10.1016/j.nicl.2012.11.003 (2012). 24179757 PMC3777837

[b48] Paulus M. P. & Stein M. B. Interoception in anxiety and depression. Brain structure & function 214, 451–463, doi: 10.1007/s00429-010-0258-9 (2010). 20490545 PMC2886901

[b49] Nunn K., Frampton I., Gordon I. & Lask B. The fault is not in her parents but in her insula—A neurobiological hypothesis of anorexia nervosa. European Eating Disorders Review 16, 355–360, doi: 10.1002/erv.890 (2008). 18711713

[b50] Jakab A., Molnar P. P., Bogner P., Beres M. & Berenyi E. L. Connectivity-based parcellation reveals interhemispheric differences in the insula. Brain topography 25, 264–271, doi: 10.1007/s10548-011-0205-y (2012). 22002490

[b51] Kurth F., Zilles K., Fox P., Laird A. & Eickhoff S. A link between the systems: functional differentiation and integration within the human insula revealed by meta-analysis. Brain Structure and Function 214, 519–534, doi: 10.1007/s00429-010-0255-z (2010). 20512376 PMC4801482

[b52] Mutschler I. *et al.* Functional organization of the human anterior insular cortex. Neuroscience letters 457, 66–70, doi: 10.1016/j.neulet.2009.03.101 (2009). 19429164

[b53] Showers M. J. & Lauer E. W. Somatovisceral motor patterns in the insula. The Journal of comparative neurology 117, 107–115 (1961). 13912292 10.1002/cne.901170109

[b54] Werner N. S. *et al.* Hippocampal function during associative learning in patients with posttraumatic stress disorder. J Psychiatr Res 43, 309–318, doi: 10.1016/j.jpsychires.2008.03.011 (2009). 18490028

[b55] Shang J. *et al.* Alterations in low-level perceptual networks related to clinical severity in PTSD after an earthquake: a resting-state fMRI study. Plos One 9, e96834, doi: 10.1371/journal.pone.0096834 (2014). 24823717 PMC4019529

[b56] Fernandez M. *et al.* Brain function in a patient with torture related post-traumatic stress disorder before and after fluoxetine treatment: a positron emission tomography provocation study. Neuroscience letters 297, 101–104, doi: 10.1016/S0304-3940(00)01674-8 (2001). 11121880

[b57] Baldaçara L. *et al.* Reduced cerebellar left hemisphere and vermal volume in adults with PTSD from a community sample. Journal of Psychiatric Research 45, 1627–1633, doi: 10.1016/j.jpsychires.2011.07.013 (2011). 21824628

[b58] Schmahmann J. D. & Caplan D. Cognition, emotion and the cerebellum. Brain : a journal of neurology 129, 290–292, doi: 10.1093/brain/awh729 (2006). 16434422

[b59] Schutter D. J. & van Honk J. The cerebellum on the rise in human emotion. Cerebellum 4, 290–294, doi: 10.1080/14734220500348584 (2005). 16321885

[b60] Buckner R. L., Krienen F. M., Castellanos A., Diaz J. C. & Yeo B. T. The organization of the human cerebellum estimated by intrinsic functional connectivity. Journal of neurophysiology 106, 2322–2345, doi: 10.1152/jn.00339.2011 (2011). 21795627 PMC3214121

[b61] Frewen P. A., Dozois D. J. & Lanius R. A. Assessment of anhedonia in psychological trauma: psychometric and neuroimaging perspectives. European journal of psychotraumatology 3, doi: 10.3402/ejpt.v3i0.8587 (2012). PMC3402136 22893841

[b62] Schmahmann J. D., Weilburg J. B. & Sherman J. C. The neuropsychiatry of the cerebellum - insights from the clinic. Cerebellum 6, 254–267, doi: 10.1080/14734220701490995 (2007). 17786822

[b63] Kipping J. A. *et al.* Overlapping and parallel cerebello-cerebral networks contributing to sensorimotor control: an intrinsic functional connectivity study. Neuroimage 83, 837–848, doi: 10.1016/j.neuroimage.2013.07.027 (2013). 23872155

[b64] O’Reilly J. X., Beckmann C. F., Tomassini V., Ramnani N. & Johansen-Berg H. Distinct and overlapping functional zones in the cerebellum defined by resting state functional connectivity. Cerebral cortex (New York, N.Y.: 1991) 20, 953–965, doi: 10.1093/cercor/bhp157 (2010). PMC2837094 19684249

[b65] Weston C. S. Posttraumatic stress disorder: a theoretical model of the hyperarousal subtype. Frontiers in psychiatry 5, 37, doi: 10.3389/fpsyt.2014.00037 (2014). 24772094 PMC3983492

[b66] Wingenfeld K. & Wolf O. T. Stress, memory, and the hippocampus. Frontiers of neurology and neuroscience 34, 109–120, doi: 10.1159/000356423 (2014). 24777135

[b67] Shin L. M. & Handwerger K. Is posttraumatic stress disorder a stress-induced fear circuitry disorder? Journal of traumatic stress 22, 409–415, doi: 10.1002/jts.20442 (2009). 19743481

[b68] Ke J. *et al.* A longitudinal fMRI investigation in acute post-traumatic stress disorder (PTSD). Acta radiologica (Stockholm, Sweden: 1987), doi: 10.1177/0284185115585848 (2015). 25995310

[b69] Weinberger D. R. & Radulescu E. Finding the elusive psychiatric “lesion” with 21st-century neuroanatomy: a note of caution. American Journal of Psychiatry 173, 27–33 (2015). 26315983 10.1176/appi.ajp.2015.15060753

[b70] Gillihan S. J. & Parens E. Should We Expect “Neural Signatures” for DSM Diagnoses? The Journal of clinical psychiatry 72, 1, 478–1389 (2011). 10.4088/JCP.10r06332gre 21457680

[b71] Shepherd A. M., Matheson S. L., Laurens K. R., Carr V. J. & Green M. J. Systematic meta-analysis of insula volume in schizophrenia. Biological psychiatry 72, 775–784, doi: 10.1016/j.biopsych.2012.04.020 (2012). 22621997

[b72] Strakowski S. M., DelBello M. P., Adler C., Cecil D. M. & Sax K. W. Neuroimaging in bipolar disorder. Bipolar disorders 2, 148–164 (2000). 11256682 10.1034/j.1399-5618.2000.020302.x

[b73] Radua J. & Mataix-Cols D. Voxel-wise meta-analysis of grey matter changes in obsessive-compulsive disorder. The British journal of psychiatry : the journal of mental science 195, 393–402, doi: 10.1192/bjp.bp.108.055046 (2009). 19880927

[b74] Radua J. *et al.* A new meta-analytic method for neuroimaging studies that combines reported peak coordinates and statistical parametric maps. European psychiatry : the journal of the Association of European Psychiatrists 27, 605–611, doi: 10.1016/j.eurpsy.2011.04.001 (2012). 21658917

[b75] Radua J., Via E., Catani M. & Mataix-Cols D. Voxel-based meta-analysis of regional white-matter volume differences in autism spectrum disorder versus healthy controls. Psychological medicine 41, 1539–1550, doi: 10.1017/S0033291710002187 (2011). 21078227

[b76] Laird A. R. *et al.* ALE meta-analysis: controlling the false discovery rate and performing statistical contrasts. Human brain mapping 25, 155–164, doi: 10.1002/hbm.20136 (2005). 15846811 PMC6871747

[b77] Wager T. D., Lindquist M. A., Nichols T. E., Kober H. & Van Snellenberg J. X. Evaluating the consistency and specificity of neuroimaging data using meta-analysis. NeuroImage 45, S210–S221, doi: 10.1016/j.neuroimage.2008.10.061 (2009). 19063980 PMC3318962

[b78] Radua J. *et al.* Multimodal meta-analysis of structural and functional brain changes in first episode psychosis and the effects of antipsychotic medication. Neuroscience and biobehavioral reviews 36, 2325–2333, doi: 10.1016/j.neubiorev.2012.07.012 (2012). 22910680

