# Supplementary material for: Altered resting-state functional activity in posttraumatic stress disorder: A quantitative meta-analysis
Source: Sci Rep. 2016 Jun 2;6:27131. doi: 10.1038/srep27131 (PMC4890007; doi:10.1038/srep27131)
Supplement: Supplementary Information [file srep27131-s1.pdf]

Altered resting-state functional activity in posttraumatic stress disorder: A quantitative meta-analysis

Ting Wang[1,2,†], Jia Liu[2,†], Junran Zhang[1,2,*], Wang Zhan[3], Lei Li[2], Min Wu[2], Hua Huang[1], Hongyan Zhu[4,*], Graham J. Kemp[5], Qiyong Gong[6,2]

[1]Department of Medical Information Engineering, School of Electrical Engineering and Information, Sichuan University, Chengdu, P.R. China. [2]Huaxi MR Research Center (HMRRC), Department of Radiology, West China Hospital of Sichuan University, Chengdu, P.R. China. [3]Neuroimaging Center, University of Maryland, College Park, Maryland, USA. [4]Laboratory of Stem Cell Biology, State Key Laboratory of Biotherapy, West China Hospital of Sichuan University, Chengdu, P.R. China. [5]Magnetic Resonance and Image Analysis Research Centre (MARIARC) and Institute of Ageing and Chronic Disease, University of Liverpool, United Kingdom. [6] Department of Psychology， School of Public Administration, Sichuan University, Chengdu, P.R. China. [†]These authors are co-first authors and contributed equally to this work. [*]Corresponding author. Department of Medical Information Engineering, School of Electrical Engineering and Information, Sichuan University, No.24, South Section One, First Ring Road, Chengdu 610065, P.R. China. Tel.: +86 13980789485. Fax: +86 028 85423503. *E-mail address:* zhangjunran@gmail.com (J.-R. Zhang), hyzhu_hmrrc@126.com (H.-Y. Zhu).

Supplementary information

Table S1. Quality assessment checklist

**Category 1: Subjects**
1. Patients evaluated prospectively; specific diagnostic criteria applied; demographic data reported.
2. Comparison subjects evaluated prospectively; psychiatric and medical illnesses excluded; demographic data reported.
3. Important confounds (e.g. age, gender, trauma type, illness duration, medication status, comorbidity, illness severity) controlled either by stratification or statistically.
4. Sample size per group > 10.
**Category 2: Methods for image acquisition and analysis**
5. Whole brain analysis automated with no apriori regional selection.
6. Coordinates reported in a standard space.
7. Imaging technique clearly enough described to be reproduced.
8. Measurements clearly enough described to be reproduced.
**Category 3: Results and Conclusions**
9. Statistical parameters provided for significant and important non-significant differences.
10. Conclusions consistent with results; limitations discussed.

When criteria were partially met, 0.5 points were assigned. The aim of this rating was to describe the completeness of published studies with a numeric score in order to aid readers, and it is not intended to critique the investigators or the work itself.
